# LncRNA XIST mediates bovine mammary epithelial cell inflammatory response via NF‐κB/NLRP3 inflammasome pathway

**DOI:** 10.1111/cpr.12525

**Published:** 2018-10-25

**Authors:** Mengru Ma, Yifei Pei, Xixi Wang, Jiaxin Feng, Yong Zhang, Ming‐Qing Gao

**Affiliations:** ^1^ College of Veterinary Medicine Northwest A&F University Yangling China; ^2^ College of Animal Science and Technology Northwest A&F University Yangling China; ^3^ Key Laboratory of Animal Biotechnology, Ministry of Agriculture Northwest A&F University Yangling China

**Keywords:** bovine, lncRNA XIST, mastitis, NF‐κB pathway, NLRP3 inflammasome

## Abstract

**Objectives:**

The correlations between long non‐coding RNAs (lncRNAs) and diverse mammal diseases have been clarified by many researches, but the cognition about bovine mastitis‐related lncRNAs remains limited. This study aimed to investigate the potential role of lncRNA X‐inactive specific transcript (XIST) in the inflammatory response of bovine mammary epithelial cells.

**Materials and methods:**

Two inflammatory bovine mammary alveolar cell‐T (MAC‐T) models were established by infecting the cells with *Escherichia coli (E. coli)* and *Staphylococcus aureus *(*S. aureus*). The expressions of pro‐inflammatory cytokines were measured, and the proliferation, viability and apoptosis of the inflammatory cells were evaluated after XIST was knocked down by an siRNA. The relationship among XIST, NF‐κB pathway and NOD‐like receptor protein 3 (NLRP3) inflammasome was investigated using an inhibitor of NF‐κB signal pathway.

**Results:**

The expression of XIST was abnormally increased in bovine mastitic tissues and inflammatory MAC‐T cells. Silencing of XIST significantly increased the expression of *E. coli* or *S. aureus*‐induced pro‐inflammatory cytokines. Additionally, knockdown of XIST could inhibit cell proliferation, suppress cell viability and promote cell apoptosis under inflammatory conditions. Furthermore, XIST inhibited *E. coli* or *S. aureus*‐induced NF‐κB phosphorylation and the production of NLRP3 inflammasome.

**Conclusions:**

The expression of XIST was promoted by activated NF‐κB pathway and, in turn, XIST generated a negative feedback loop to regulate NF‐κB/NLRP3 inflammasome pathway for mediating the process of inflammation.

## 1 INTRODUCTION

Bovine mastitis continues to be one of the most common diseases worldwide so far and has been seriously impacting on milk yield, milk composition and animal welfare.^1^ Mammary gland inflammation is mainly caused by the interaction of the host, pathogens and environmental factors.^2^ *Escherichia coli* (*E. coli*) and *Staphylococcus aureus* (*S. aureus*) are two of the major contagious pathogens which account for clinical and subclinical bovine mastitis through rapid multiplication and persistent adhesion in mammary gland tissue.^3^ In bovine mastitis tissue, Toll‐like receptors (TLR)‐mediated transduction elicits a cascade of immune responses.^4^ Meanwhile, these inflammatory mammary glands display a series of inflammatory characteristics, such as tissue damage, cell death, cytokine production,^5^ changes in cell proliferation and migration,^6^ etc

Several signalling pathways are involved in inflammatory reaction, and nuclear factor‐κB (NF‐κB) is considered as a vital one. Activated NF‐κB contributes to regulating the process of cell proliferation and apoptosis, and the production of inflammatory cytokines including tumour necrosis factor (TNF)‐α, interleukin (IL)‐1β, IL‐6 and IL‐8.^7^, ^8^ In addition, current studies have shown that activated NF‐κB pathway could function as an upstream activator of the NOD‐like receptor (NLR) family member pyrin domain‐containing protein 3 (NLRP3), which is known as the inflammasome consisted of NLRP3, ASC and serine protease caspase‐1. Inflammasomes are cytoplasmic multiprotein complexes, including four individual inflammasome branches (NLRP1, NLRP3, NALP4 and AIM2).^9^, ^10^ Inflammasomes can be generated and activated by various stimulators,^11^ and *E. coli* and *S. aureus* identified in bovine mastitis are considered as main stimuli.^12^, ^13^ A few studies have reported that the complex network of NF‐κB and NLRP3 inflammasome played an important role in mastitis,^14^ but the specific mechanism between NF‐κB and NLRP3 inflammasome needs to be further identified.

Long non‐coding RNAs (LncRNAs) are defined as a novel class of transcripts with more than 200 nucleotides in length that generally lack the capability of coding protein and that were once regarded as transcriptional noises.^15^ Increasing evidences have indicated that lncRNAs exert crucial functions in gene regulation, biological process and several diseases, such as inflammation^16^ and immune response.^17^ LncRNA X inactivate‐specific transcript (XIST), a 17‐kb‐long RNA transcribed by the inactive X chromosome, is thought to be involved in the X chromosome inactivation in female mammals, inducing the compensation for X‐linked gene dosage imbalance between the sexes.^18^, ^19^ More recently, accumulating evidences suggested that the expression of XIST was up‐regulated in diverse cancer types including breast cancer,^20^ gastric cancer,^21^ lung cancer^22^ and cystic fibrosis,^23^ involving in cell proliferation, migration, invasion as well as apoptosis.^24^ As the microenvironment of tumour is similar to the inflammatory condition,^25^ XIST may also play a potential role in inflammatory process.

This study aimed to investigate whether XIST played a critical role in regulating the inflammatory response to pathogenic stimulus through NF‐κB pathway and further identify the relationship between NF‐κB pathway and NLRP3 inflammasome in bovine mastitis.

## 2 MATERIALS AND METHODS

### 2.1 Tissue specimens, cell line culture and cell transfection

Normal and mastitic mammary tissues used in this study were stored at −80°C in our laboratory before use, and the tissue collection procedure was described in our previous publication.^26^ The bovine mammary alveolar cell‐T (MAC‐T) cell line was a gift from Prof. Mark D. Hanigan (Virginia Polytechnic Institute and State University, Blacksburg, VA). The cell line was routinely cultured in completed medium (DMEM/F12; Life Technology, Burlington, VT) with 10% foetal bovine serum (Gibco BRL, Grand Island, NY), 100 IU/mL penicillin (Gibco BRL) and 100 μg/mL streptomycin (Gibco BRL) added in.

Transfection of small interfering RNA (siRNA) was conducted using the lipofectamine 2000 (GenePharama, Shanghai, China) according to the manufacturer's protocol. An siRNA targeting bovine XIST (Si‐XIST) and a negative control siRNA (Si‐NC) were purchased from GenePharama. After 6 hours of transfection, the residual siRNA/lipofectamine 2000 mix was replaced with fresh medium. The transfected cells were cultured for another 12 hours before subsequent experiments. The primer sequences of the siRNAs were as follows: Si‐XIST, 5′‐ GAC CUU GUC AUG UGG AUA UTT −3′ (forward) and 5′‐ AUA UCC ACA UGA CAA GGU CTT −3′ (reverse); Si‐NC, 5′‐ UUC UCC GAA CGU GUC ACG UTT −3′ (forward) and 5′‐ ACG UGA CAC GUU CGG AGA ATT −3′ (reverse).

To inhibit the NF‐κB pathway, the MAC‐T cells were treated with BAY 11‐7083 (Beyotime, Shanghai, China) at a final concentration of 3 µmol/mL. When the incubation time of BAY 11‐70831 arrived at 1 hour, the *E. coli* or *S. aureus* was added to stimulate the cells for subsequent experiments.

### 2.2 Stimulation of MAC‐T cells with inactivated *Escherichia* *coli* and *Staphylococcus* *aureus*

*Escherichia coli* strain ATCC29213 and *S. aureus* strain ATCC25922 were obtained from the American Type Culture Collection (Manassas, VA). Bacteria were grown in Luria‐Bertain (LB) broth at 37°C overnight, and the bacterial suspensions were washed by phosphate‐buffered saline (PBS) for 3s times. Next, the bacteria were diluted in PBS to a series of concentration gradients. For each dilution, 10 µL was put onto LB agar medium overnight. The density of bacterial suspension was calculated by the colony count. Meanwhile, *E. coli* and *S. aureus* cells were killed by heating for 1 hour at 65°C and 2 hours at 60°C, respectively. Successful inactivation was verified when no bacteria colony was observed after overnight incubation on LB at 37°C.

The MAC‐T cells were seeded into 6‐well plates overnight at 37°C. Then, the cells were stimulated with inactive *E. coli* or *S. aureus*. The ratio of MAC‐T cells to *E. coli* and *S. aureus* was 1:1000 and 1:100, respectively. After 24 hours of stimulation, the MAC‐T cells were harvested for subsequent analyses.

### 2.3 RNA extraction and Real‐time quantitative PCR

The total RNA from the mammary tissues or cells was extracted using TriZol solution (TransGene, Shanghai, China) according to the manufacturer's instructions. The assessment of the quantity and quality of the total RNA was conducted by a spectrophotometer (NanoDrop Technologies, Wilmington, DE). The first‐strand cDNA was generated from 3 µg total RNA using TransScript II First‐Strand cDNA Synthesis SuperMix (TransGene). Real‐time quantitative PCR (RT‐qPCR) was carried out to examine the levels of genes by iQ5 light cycler (Bio‐Rad, Hercules, CA) in 20 µL reactions. Finally, the expression of each gene was normalized to GAPDH. The primers used are listed in Table S1.

### 2.4 Western blot analysis

The MAC‐T cells were washed 3 times with PBS for 5 minutes and lysed with 150 μL PRO‐PREP Protein Extraction Solution (iNtRON Biotechnology, Inc, Gyeonggi‐do, Korea) per well on ice to obtain the total protein. Protein concentrations were measured using Bradford Easy Protein Quantitative Kit (TransGene). Equal amount of protein samples were separated using 5%‐10% SDS‐PAGE with running buffer and transferred onto polyvinylidene fluoride membranes (Millipore, Bedford, MA). Then, the blots were incubated with antibody against: anti‐NLRP3 (Proteintech, Wuhan, China), anti‐ASC (Proteintech), anti‐pro‐caspase‐1 (Proteintech), p65 (Santa Cruz, Dallas, TX), pp65 (Bioss, Beijing, China), IκB (Bioss), pIκB (Cell Signaling Technology, Boston, MA) and anti‐GAPDH (TransGene) antibodies at 4°C overnight. After washing with TBST, the membranes were then incubated with HRP‐conjugated secondary antibodies (Beyotime). Finally, immunoreactive proteins were detected and quantified by an enhanced chemiluminescence solution (Beyotime).

### 2.5 Cell viability assay

Cell viability was performed using Cell Counting Kit‐8 (TransGene). Briefly, cells were plated in 96‐well plates at the same density of 2 × 10^3 ^cells/well and cultured for 0, 24, 48 and 72 hours. At the indicated timepoint, CCK‐8 solution at a medium dilution of 1:10 diluted was added to each well and the plate was incubated at 37°C for 3 hours. The absorbance was measured by a microplate reader (Bio‐Rad, Hercules, CA) at a wavelength of 450 nm, and the proliferation of each groups was calculated using the equation: [(OD_n_−OD_blank_) − (OD_0_−OD_blank_)]/(OD_0_−OD_blank_) × 100. In this formula, OD_n_ means the absorbance value at the indicated day and OD_0_ means the absorbance value at 0 day after culture, while OD_blank_ refers to the absorbance value of the wells without cells.

### 2.6 Cell proliferation assay

Cell proliferation was analysed by 5‐ethynyl‐2′‐deoxyuridine (EdU) cell proliferation Assay Kit (Ribobio, Guangzhou, China). In brief, cells were cultured in 48‐well plates for 12 hours and then transfected with Si‐XIST or Si‐NC. After another 12 hours, Si‐XIST or Si‐NC silenced cells were infected with inactive *E. coli* or *S. aureus* for 24 hours. Then, the cells were incubated with EdU solution for 2 hours at 37°C. The labelled cells were fixed with 4% paraformaldehyde for 20 minutes and then incubated with glycine. Subsequently, cells were treated with 0.5% Triton‐X100 for 10 minutes and washed with PBS. After that, cells were stained with Apollo for 30 minutes in dark. Followed by staining with Hoechst‐33342 for another 30 minutes, the proportion of cells was finally shown as the ratio of the fluorescent positive cells to total labelled cells.

### 2.7 Flow cytometry

The percentage of apoptosis cells was assessed by Flow cytometry analysis with a commercial apoptosis assay kit (TransGene). Cells were collected and washed three times by cold PBS and then were double strained with Annexin V/PI according to the manufacture's instructions. After staining, the cells were analysed by flow cytometer. Finally, viable cells, early stage apoptotic cells, late stage apoptotic cells and death cells were determined with WinMDI software.

### 2.8 Enzyme‐linked immunosorbent assay

The protein secretion level of TNF‐α, IL‐6, IL‐1β and IL‐18 in each group was measured by enzyme‐linked immunosorbent assay (ELISA). MAC‐T cells at 70% confluence were transfected with either Si‐NC or Si‐XIST for 12 hours and then cultured in a fresh medium before MAC‐T cells were treated with inactive *E. coli* or *S. aureus*. Finally, the culture supernatant of each groups was collected and the secretions of TNF‐α, IL‐6, IL‐1β and IL‐18 were measured by their corresponding ELISA kits (Huzhen Biological Technology, Shanghai, China), according to the manufacturer's instructions.

### 2.9 Luciferase assays

MAC‐T cells were seeded in 48‐well culture dishes for 12 hours and then co‐transfected with siRNA, NF‐κB luciferase plasmids (Promega, Madison, WI) and Renilla luciferase plasmids (Promega) using Lipofectamine2000 according to the manufacturer's protocol. After 12 hours of transfection, MAC‐T cells were stimulated with inactive *E. coli* or *S. aureus* for another 24 hours. Finally, cells were lysed and the activity of luciferase was analysed using Dual‐Luciferase Reporter Assay System (TransGene).

### 2.10 Immunofluorescence analysis

MAC‐T cells were transfected with either Si‐NC or Si‐XIST for 12 hours and then stimulated with inactive *E. coli* or *S. aureus* for another 24 hours. After pretreatment, MAC‐T cells were fixed with 4% paraformaldehyde for 15 minutes and then permeabilized with 0.5% Triton X‐100 (Beyotime) for 10 minutes. Subsequently, MAC‐T cells were blocked with immunofluorescence sealing fluid (Beyotime) and incubated with anti‐p65 antibody at 4°C overnight. After washing with PBS, MAC‐T cells were incubated with secondary Alexa Fluor 488‐labelled goat anti‐rabbit IgG antibody (Bioss) for 2 hours at room temperature. Subsequently, nuclei were stained with DAPI (Beyotime) for 10 minutes at room temperature. Finally, immunofluorescence was then visualized under an immunofluorescence microscope.

### 2.11 Statistical analysis

The statistical data were analysed with the Statistical Product and Service Solutions (SPSS 19.0 for Windows; IBM). One‐way ANOVA test was used to determine the differences. Unless otherwise specified, the data were expressed as mean values ±standard deviation (SD). *P < 0.05 and **P < 0.01 was considered statistically significant. Each treatment was repeated at least three times.

## 3 RESULTS

### 3.1 LncRNA XIST was up‐regulated in bovine mastitic tissues and in inflammatory bovine mammary epithelial cell models

**Figure 1:**
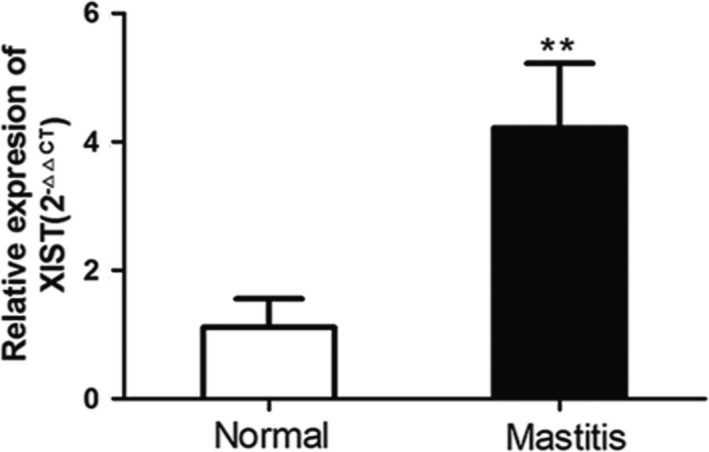
The expression levels of XIST in normal and mastitic samples of bovine mammary glands. XIST genic expression levels in normal and mastitic tissues of bovine mammary glands (n = 4) were analysed by RT‐qPCR (mean ± SD). **P < 0.01 vs normal

To explore the potential function of XIST during the process of bovine mastitis, we firstly performed RT‐qPCR analysis to measure the expression level of lncRNA XIST in bovine mammary tissues. We found lncRNA XIST was significantly up‐regulated in bovine mastitic tissues compared with counterpart normal tissues (Figure S1 and Figure 1).

**Figure 2:**
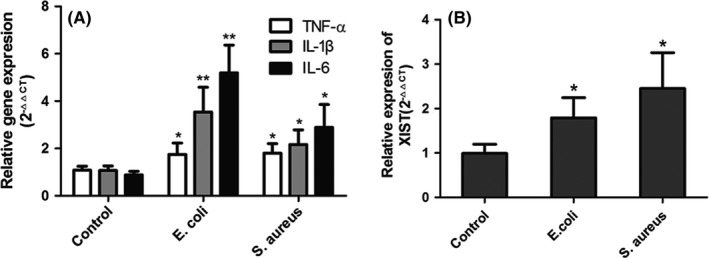
The expression levels of XIST in MAC‐T cells with and without *Escherichia coli* (*E. coli*) or *Staphylococcus aureus* (*S. aureus*) treatment. A, Two inflammatory MAC‐T cell models were established by *E. coli* and *S. aureus* stimulation in vitro. The mRNA levels of inflammatory biomarkers (TNF‐α, IL‐1β and IL‐6) were quantified by RT‐qPCR. B, The expression levels of XIST in treated and untreated MAC‐T cells were analysed by RT‐qPCR. All data were presented as the mean ± SD (n = 3). *P < 0.05, **P < 0.01 vs control

In addition, two inflammatory MAC‐T cell models of bovine mastitis were established by infecting the cells with heat‐killed *E. coli* and *S. aureus*, because these two heat‐inactivated bacteria could induce the similar immune responses as live *E. coli* and *S. aureus*.^27^, ^28^ The expression levels of three pro‐inflammatory cytokines (TNF‐α, IL‐6 and IL‐1β) were significantly up‐regulated (Figure 2A), indicating a successful establishment of bovine mastitis cell model. Consistent with the expression trend in bovine mastitic tissues, lncRNA XIST expression level was also significantly higher in inflammatory MAC‐T cells than that in normal MAC‐T cells (Figure 2B). These results indicated that increased expression of XIST was critically involved in the process of bovine mastitis.

### 3.2 **Knockdown of XIST intensified *Escherichia*** ***coli *or *Staphylococcus*** ***aureus*‐induced inflammatory response in MAC‐T cells**

**Figure 3:**
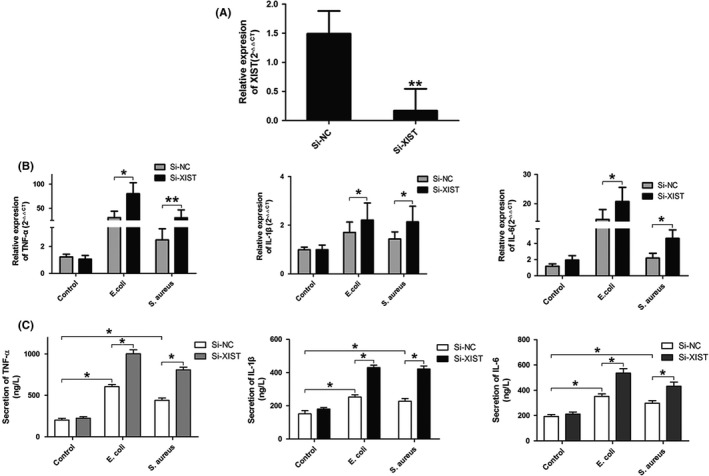
The expression levels of pro‐inflammatory cytokines in XIST knockdown cells. A, An siRNA was designed for XIST (Si‐XIST) gene loci and the knockdown efficiency was detected by RT‐qPCR. A non‐targeting siRNA (Si‐NC) was used as control. B and C, Gene and protein expression levels of TNF‐ɑ, IL‐1β and IL‐6 in *Escherichia coli* or *Staphylococcus aureus*‐induced inflammatory MAC‐T cells transfected with Si‐NC or Si‐XIST were analysed by RT‐qPCR and ELISA, respectively. All data were presented as the mean ± SD (n = 3). *P < 0.05, **P < 0.01

Considering that XIST was up‐regulated in MAC‐T cells in response to *E. coli* or *S. aureus* infection, we wondered whether XIST was involved in the regulation of *E. coli* or *S. aureus*‐triggered production of pro‐inflammatory factors through potential mechanisms in bovine mastitis. To investigate the functional role of XIST, Si‐XIST was designed and transfected in MAC‐T cells to down‐regulate XIST. An efficient transfection of Si‐XIST was observed under a fluorescent microscope (Figure S2), and the XIST expression level was significantly down‐regulated (over than 90%) in the cells transfected with Si‐XIST compared with that in those cells transfected with Si‐NC (Figure 3A). Furthermore, the pro‐inflammatory cytokines of TNF‐ɑ, IL‐1β and IL‐6 were significantly higher in inflammatory MAC‐T cells after XIST was knocked down (Figure 3B,C). These results suggested that XIST could regulate the expression of inflammatory cytokines and modulate the *E. coli* or *S. aureus*‐triggered inflammatory response in MAC‐T cells.

### 3.3 XIST mediated cell proliferation, viability and apoptosis of inflammatory MAC‐T cells

**Figure 4:**
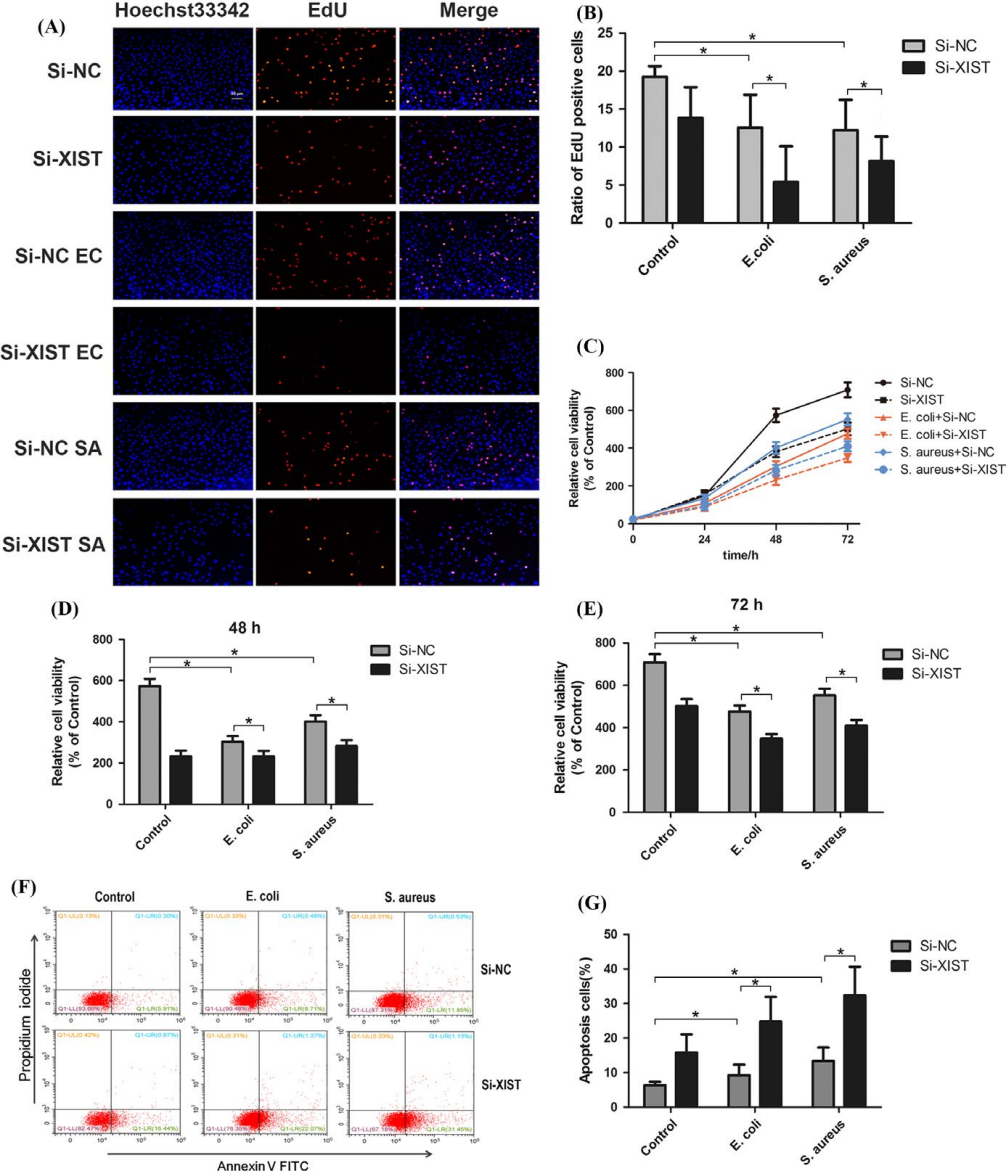
The effect of XIST knockdown on cell proliferation, viability and apoptosis of MAC‐T cells under inflammatory conditions. A and B, After 48 hours of transfection with Si‐XIST and Si‐NC, a 5‐ethynyl‐2′‐deoxyuridine (EdU) assay was performed on inflammatory MAC‐T cells. EdU incorporation rate was calculated as the ratio of EdU positive cells (red) relative to Hoechst 33342 positive cells (blue). C, D and E, Cell viability of inflammatory MAC‐T cells was assessed by CCK‐8 assay after being transfected with Si‐XIST or Si‐NC. F and G, Annexin V‐FITC/PI staining was detected by flow cytometry. Inflammatory MAC‐T cells were transfected with Si‐XIST or Si‐NC. The apoptosis ratio was the sum of the early and late apoptosis cell percentage. All data were presented as the mean ± SD (n = 3). *P < 0.05. Abbreviations: Si‐NC, a negative control siRNA; Si‐XIST, an siRNA targeting bovine XIST; ‐EC and ‐SA, MAC‐T cells were stimulated with *Escherichia coli *and *Staphylococcus aureus*

The cell proliferation was evaluated by EdU incorporation assay. The results showed the number of EdU positive cells in *E. coli *or *S. aureus‐*treated group was lower than that in control group, indicating the cell proliferation ability was suppressed after MAC‐T cells were stimulated with *E. coli* or *S. aureus*. Moreover, in both *E. coli* and *S. aureus‐*treated group, knockdown of XIST significantly decreased the number of EdU positive cells, indicating that silencing of XIST suppressed the proliferation ability of inflammatory MAC‐T cells (Figure 4A,B).

CCK‐8 assay was carried out to examine the viability of cells. The viability of MAC‐T cells infected with *E. coli* or *S. aureus* were lower than that of the normal MAC‐T cells, and silencing of XIST could further decrease cell viability in the two inflammatory MAC‐T cell groups compared with their counterparts (Figure 4C‐E).

Cell apoptosis was analysed by Annexin V/PI staining, followed by subsequent flow cytometry analysis. We found that the percentages of apoptotic cells was significantly increased in the inflammatory MAC‐T cell groups compared with the normal MAC‐T cell group, and silencing of XIST enhanced the apoptosis rate of the inflammatory cells compared with those transfected with Si‐NC (Figure 4F,G), indicating that knockdown of XIST could promote the apoptosis of MAC‐T cells under inflammation conditions.

### 3.4 XIST suppressed the activation of NF‐κB signalling pathway and reduced the production of NLRP3 inflammasome

**Figure 5:**
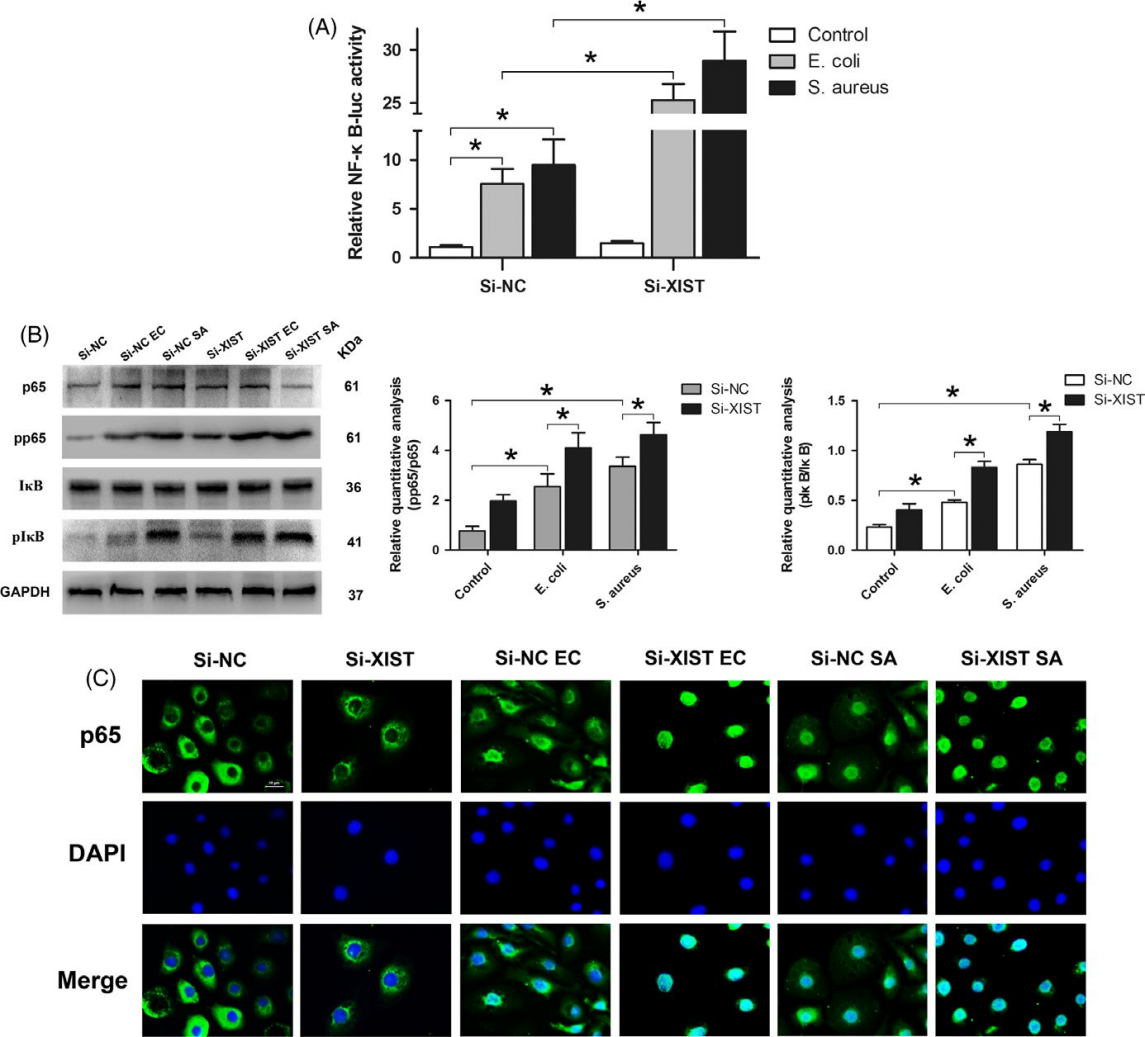
The effect of XIST on NF‐κB signal pathway. A, The MAC‐T cells were co‐transfected with siRNA, NF‐κB luciferase plasmids and Renilla luciferase plasmids, and then stimulated with *Escherichia coli *or *Staphylococcus aureus*. The luciferase activity of the NF‐κB reporter was measured by Dual‐Luciferase Reporter Assay System and the activity of firefly luciferase was normalized to that of Renilla luciferase. B, The activations of two key signal subunits of the NF‐κB pathway (p65 and IκB) were analysed by Western blot and subsequent quantitative analysis. GAPDH was used as a loading control. C, Immunofluorescent staining was conducted to show the localization of NF‐κB p65 subunit (Green). The nuclei were coloured with DAPI (blue). All data were presented as the mean ± SD (n = 3). *P < 0.05. Abbreviations: Si‐NC, a negative control siRNA; Si‐XIST, an siRNA targeting bovine XIST; ‐EC and ‐SA, MAC‐T cells were stimulated with *Escherichia coli *(*E coli*) and *Staphylococcus aureus *(*S aureus*); pp65, phosphorylated p65; pIκB, phosphorylations of IκB

As XIST could regulate the expression of pro‐inflammatory cytokines, we speculated that XIST might be involved in the activation of upstream NF‐κB pathway, a well‐known inflammation‐related signal pathway, and its target genes. To confirm our speculation, we firstly performed dual‐luciferase reporter assays to examine the activation of NF‐κB. Results showed that knockdown of XIST significantly enhanced the activity of the NF‐κB luciferase reporter gene under inflammatory conditions (Figure 5A). In addition, we found the phosphorylations of p65 and IκB had a significant increase in the inflammatory MAC‐T cells transfected with Si‐XIST compared with those transfected with Si‐NC (Figure 5B). Moreover, immunofluorescence staining showed that knockdown of XIST could promote the translocation of NF‐κB p65 subunit from cytoplasm to nucleus in *E. coli* or *S. aureus*‐induced inflammatory MAC‐T cells (Figure 5C). These results suggest that *E. coli* or *S. aureus*‐triggered activation of NF‐κB pathway was suppressed by XIST.

**Figure 6:**
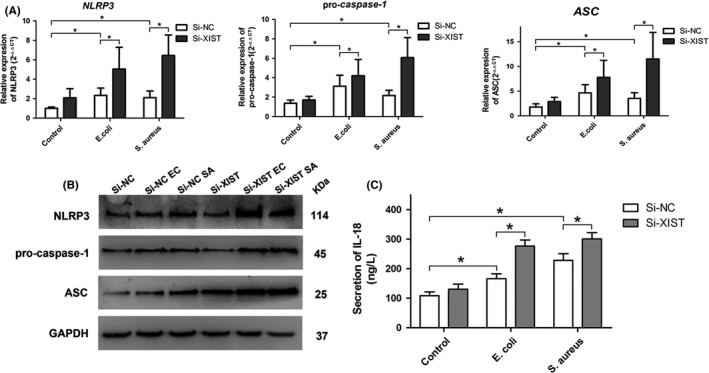
The effect of XIST on the expression of NLRP3 inflammasome. A and B, Gene and protein expression levels of three NLRP3 inflammasome components (NLRP3, pro‐casepase‐1 and ASC) in MAC‐T cells after transfection with Si‐NC or Si‐XIST and then stimulated with *Escherichia coli* (*E. coli*) or *Staphylococcus aureus* (*S. aureus*) were quantified by RT‐qPCR and western blot, respectively. C, The secretion level of NLRP3 pathway end product (IL‐18) was measured by ELISA. All data were presented as the mean ± SD (n = 3). *P < 0.05. Abbreviations: Si‐NC, a negative control siRNA; Si‐XIST, an siRNA targeting bovine XIST; ‐EC and ‐SA, MAC‐T cells were stimulated with *E. coli* and *S. aureus*

The NLRP3 inflammasome has been demonstrated to play a crucial role in the process of inflammation of mammary glands.^29^ To elucidate whether the anti‐inflammatory mechanism of XIST was associated with NLRP3 inflmmasome, we further examined the levels of NLRP3 inflmmasome components, including NLRP3, pro‐casepase‐1 and ASC, in inflammatory MAC‐T cells. The results showed that *E. coli* or *S. aureus* was able to induce the formation of NLRP3 inflammasome, as indicated by the increased mRNA (Figure 6A) and protein (Figure 6B) expressions of NLRP3, ASC and pro‐caspase‐1, and their expressions were further increased in inflammatory MAC‐T cells transfected with Si‐XIST compared with the counterparts. In addition, the protein secretion level of NLRP3 inflammasome end products (IL‐1β and IL‐18) was significantly increased in XIST knockdown cells stimulated with *E. coli* or *S. aureus* (Figures 3C and 6C). These results suggested that XIST was able to inhibit *E. coli* or *S. aureus*‐induced production of NLRP3 inflammasome.

### 3.5 XIST mediated inflammatory response through the NF‐κB/NLRP3 inflammasome signalling pathway in MAC‐T cells

**Figure 7:**
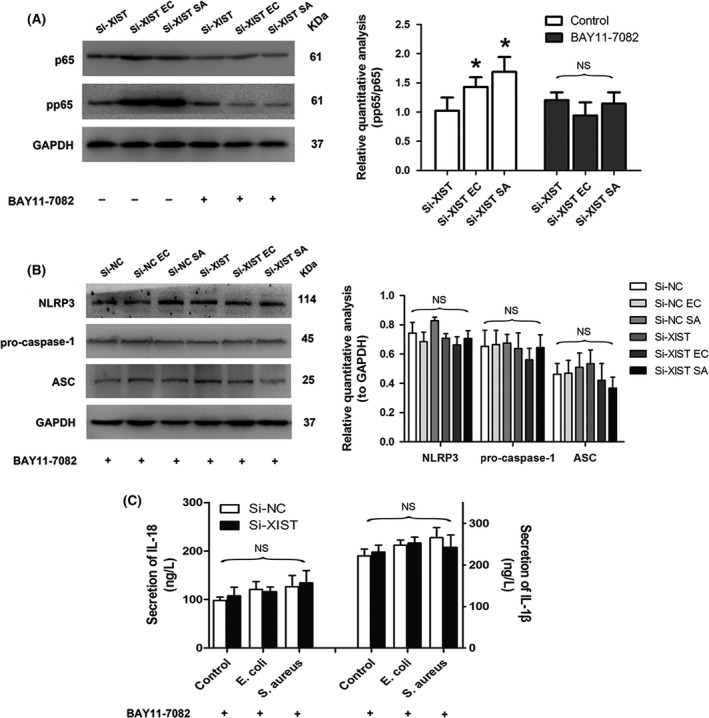
*Escherichia coli* (*E. coli*) or *Staphylococcus aureus* (*S. aureus*)‐induced NLRP3 inflammasome expression was associated with the activation of NF‐κB. A, After transfection with Si‐XIST, the MAC‐T cells were treated with the indicated concentration of BAY 11‐7082 for 1 hour, and then stimulated with *E. coli* or *S. aureus*. The effect of BAY 11‐7082 on the expression of phosphorylated p65 was measured by Western blot and subsequent quantitative analysis. B, The effects of NF‐κB inhibitor (BAY 11‐7082) on the expressions of NLRP3, pro‐caspase‐1 and ASC were measured by Western blot and subsequent quantitative analysis. GAPDH was used as the loading control. C, The protein secretion of IL‐1β and IL‐18 was measured by ELISA. *P < 0.05 vs control, NS means no significance among all columns in each group. Abbreviations: Si‐NC, a negative control siRNA; Si‐XIST, an siRNA targeting bovine XIST; ‐EC and ‐SA, MAC‐T cells were stimulated with *E. coli* and *S. aureus*; pp65, phosphorylated p65

**Figure 8:**
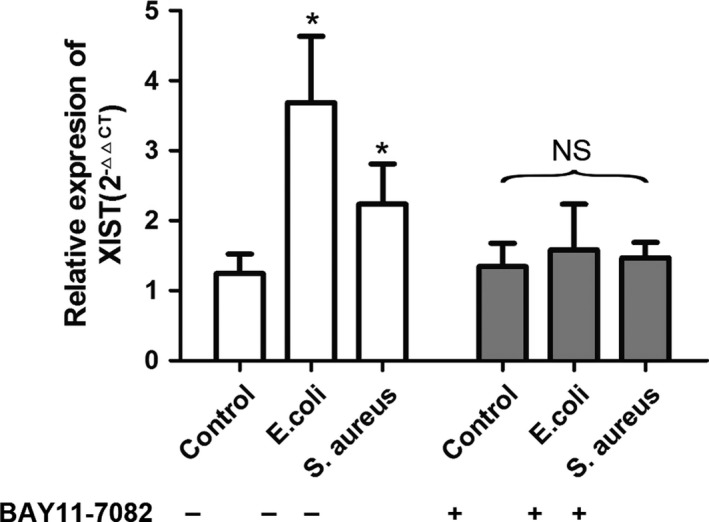
*Escherichia coli* or *Staphylococcus aureus*‐induced XIST transcription depended on NF‐κB pathway. The expression levels of XIST in MAC‐T cells were measured by RT‐qPCR under inflammatory conditions with or without the NF‐κB inhibitor (BAY 11‐7082). All data were presented as the mean ± SD (n = 3). *P < 0.05 vs control, NS means no significance among all columns

The above data showed that silencing of XIST could activate NF‐κB pathway and enhance the production of NLRP3 inflammasome in *E. coli* or *S. aureus*‐treated MAC‐T cells. We further explored the relationship among XIST, NF‐κB and NLRP3 inflammasome using an inhibitor of NF‐κB signal pathway, BAY 11‐7082. The data showed that BAY 11‐7082 significantly blocked *E. coli* or *S. aureus*‐induced phosphorylation of NF‐κB p65 in inflammatory MAC‐T cells transfected with Si‐XIST (Figure 7A). In addition, the increased expressions of NLRP3, ASC and pro‐caspase‐1 after infection with *E. coli* or *S. aureus* was obstructed in the presence of BAY 11‐7082 (Figure 7B). The increased secretion levels of NLRP3 inflammasome end products (IL‐1β and IL‐18) induced by *E. coli* or *S. aureus* were also blocked in XIST knockdown cells (Figure 7C). Furthermore, inhibiting the activation of the NF‐κB pathway also blocked *E. coli* or *S. aureus*‐induced up‐regulation of XIST in MAC‐T cells (Figure 8). These date suggested that the production of NLRP3 inflammasome and the transcription activation of XIST triggered by *E. coli* or *S. aureus* were the consequences of the activation of NF‐κB signalling pathway.

## 4 DISCUSSION

Gram‐negative *E. coli* and gram‐positive *S. aureus* are two of the most common pathogens that often result in different types of bovine mastitis during both lactation and non‐lactating periods. Previous studies have described that *E. coli* and *S. aureus* could evoke a cascade of immune responses via being recognized by TLRs.^30^ Besides, heat‐inactivated *E. coli* could increase the expression of both TLR2 and TLR4, while heat‐inactivated *S. aureus* could only promote the expression of TLR2 in bovine mammary epithelial cells.^31^ Subsequently, TLRs signal transduction pathways trigger signalling cascades in the early innate immune response and eventually activate NF‐κB factors,^32^ as well as the NLRP3 inflammasome.^33^ Successfully activated NF‐κB augments the production of downstream pro‐inflammatory cytokines such as pro‐IL‐1β, IL‐6 and TNF‐α through binding to their promoter regions.^34^ Then, the activated molecular platform NLRP3 is responsible for the formation of the active secreted protein proteolytic caspase‐1, which cleaves pro‐IL‐1β into mature IL‐1β.^35^ Therefore, these pro‐inflammatory cytokines are triggered by NF‐κB/NLRP3 inflammasome signalling pathway and then generate an inflammation microenvironment to medicate cell proliferation, viability and apoptosis. In this study, we demonstrated that treatment with inactive *E. coli* or *S. aureus* led to the activation of NF‐κB and NLRP3 inflammasome, and promoted the transcriptional activity of IL‐1β, IL‐6 and TNF‐α in bovine mammary epithelial cells. Besides, we confirmed that XIST mediated bovine epithelial cell immune response via NF‐κB/NLRP3 inflammasome pathway under inflammatory conditions.

Although lncRNAs have been shown to be important regulators in diverse pathological processes and their potential mechanisms in immunoregulation have been emerging,^36^ the exact mechanisms of lncRNAs in bovine mastitis remain mysterious. Current researches have indicated that XIST is dysregulated in a variety of female cancers. XIST is associated with breast cancer by interacting with BRCA1.^37^ In addition, the absence of XIST expression is due to the loss of Barr body in ovarian cancer.^38^ However, whether XIST serves as a mediator in such sex‐related diseases like bovine mastitis is still unknown. In this study, we found that XIST was aberrantly up‐regulated in both bovine mastitic tissues and inflammatory mammary epithelial cells. What's more, we put forward that XIST could interact with NF‐κB/NLRP3 inflammation signalling pathway and mediate the expressions of pro‐inflammatory cytokines, slowing down cell apoptosis, maintaining cell viability and promoting cell proliferation in the inflammatory process.

By exploring the function of XIST, we found that silencing of XIST could regulate a subset of genes that were involved in local inflammation. These evidences demonstrated that XIST might restrain the pathogenesis of inflammation through inhibiting the overexpressed pro‐inflammatory cytokines associated with the immune dysregulation. Previous studies have shown that the pro‐inflammatory cytokines (IL‐1β, IL‐6 and TNF‐α) could serve as mediators in cell proliferation, viability and apoptosis,^39^, ^40^, ^41^ and these prolonged and uncontrolled protein could also cause cell and tissue damages.^42^ In light of the fact that XIST could inhibit cell apoptosis and promote cell proliferation and viability under inflammatory conditions, these inflammatory factors might be important bridges between them. To gain insight of the function of XIST on *E. coli* or *S. aureus*‐induced signalling, we investigated whether the intracellular signalling pathways of NF‐κB and NLRP3 inflammasome were regulated by XIST, and we found that silencing of XIST could enhance *E. coli* or *S. aureus*‐induced NF‐κB phosphorylation, as well as the activation of NLRP3. It is well known that NLRP3 inflammasome functions as an inflammatory factor in generating inflammation by maturating IL‐1β and IL‐18.^43^ In the present study, knockdown of XIST increased the expression of NLRP3 inflammasome components (NLRP3, ASC and pro‐caspase‐1) under inflammatory conditions. Then, we further found knockdown of XIST could promote NLRP3 inflammasome end products IL‐1β and IL‐18 secretion. These results suggested that *E. coli* or *S. aureus*‐induced activation of NLRP3 inflammasome was inhibited by XIST. In addition, NLRP3 inflammasome is involved in the process of NF‐κB‐mediated inflammation.^44^ We further found that the blockade of NF‐κB pathway could reduce NLRP3 inflammasome component expression under the inflammatory condition. Therefore, these results suggested that the function of XIST in the process of *E. coli* or *S. aureus*‐induced mastitis was to prevent the production of inflammatory cytokines, particularly, through inhibiting the activation of NF‐κB. Besides, we also demonstrated that the production of NLRP3 inflammasome was based on the activation of NF‐κB pathway in the inflammatory bovine mammary epithelial cells.

**Figure 9:**
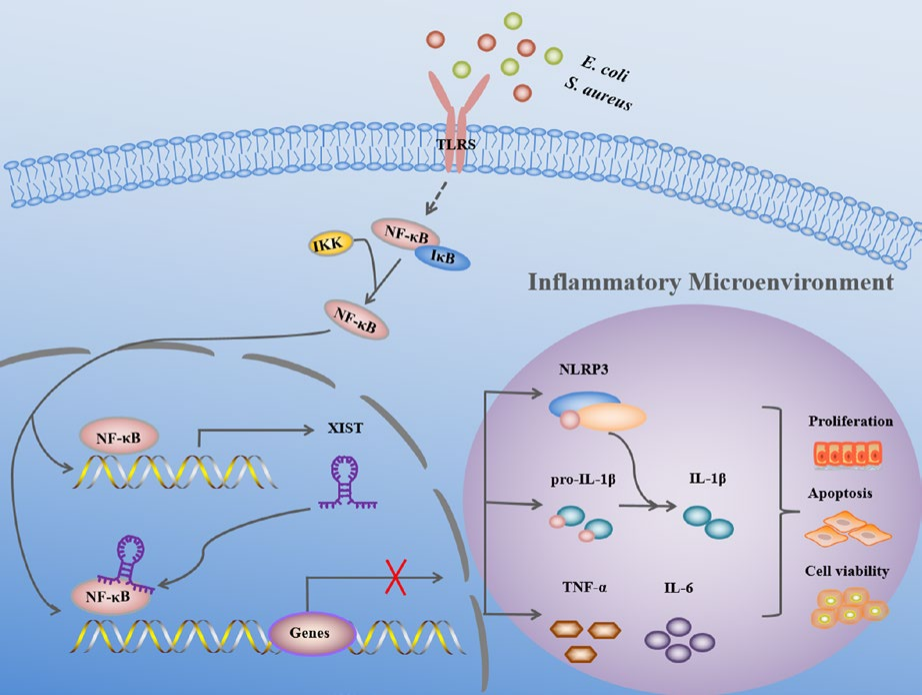
A proposed model depicting the possible mechanism of XIST on mediating cell proliferation, viability and apoptosis via generating a negative feedback regulation of NF‐κB/NLRP3 inflammasome pathway. The recognition of *Escherichia coli* or *Staphylococcus aureus* by Toll‐like receptors (TLRS) triggered the activation of NF‐κB signalling, thus stimulating the production of inflammatory genes such as TNF‐α, pro‐IL‐1β, IL‐6 and NLRP3 inflammasome. These inflammatory genes constituted an inflammatory microenviroment to modulate cell proliferation, viability and apoptosis. In turn, NF‐κB‐dependent XIST interacted with NF‐κB signal pathway and inhibited the overexpression of downstream inflammatory genes

Interestingly, our data showed that inhibition of NF‐κB pathway could abate *E. coli* or *S. aureus*‐induced up‐regulation of XIST in MAC‐T cells, suggesting that increased XIST expression was associated with NF‐κB pathway. It is reported that nuclear lncRNAs usually have the function of regulating transcription by influencing transcription factors activity.^45^ XIST was mainly located in nuclear, so it might regulate transcription in the same way as nuclear lncRNAs. Based on our data, we concluded that the interaction of XIST and NF‐κB might generate a nuclear RNA–protein complex and the NF‐κB‐controlled downstream genes were then regulated by it (Figure 9).

In conclusion, we have shown for the first time that lncRNA XIST expression was up‐regulated in bovine mastitic tissues and inflammatory mammary epithelial cells induced by bacteria in vitro. XIST promoted cell proliferation, maintained cell viability and inhibited cell apoptosis under inflammatory conditions through inhibiting the activation of NF‐κB pathway and the formations of pro‐inflammatory cytokines. Furthermore, activated NF‐κB pathway could promote the expressions of both XIST and NLRP3 inflammasome, and in turn, XIST generated a negative feedback loop to regulate the NF‐κB/NLRP3 inflammasome pathway for mediating the process of inflammation. Bovine mastitis is a localized defence response of the body components against the pathogens, but excessive and sustained inflammation leads to cell and tissue damage. So, the function of XIST in bovine mastitis was to inhibit this excessive and sustained inflammatory process. Understanding the key role of lncRNA XIST in bovine mammary epithelial cells under inflammatory conditions will contribute to the identification of new therapeutic targets to treat bovine mastitis.

## CONFLICT OF INTERESTS

The authors declare no conflict of interest.

## AUTHOR CONTRIBUTIONS

Ma and Gao performed study design. Ma and Pei performed data collection. Gao and Zhang involved in contribution of new reagents or analytical tools. Ma, Pei, Wang and Feng performed data analysis. Ma, Pei and Gao involved in manuscript preparation.
