# Supplementary material for: LncRNA XIST mediates bovine mammary epithelial cell inflammatory response via NF‐κB/NLRP3 inflammasome pathway
Source: Cell Prolif. 2018 Oct 25;52(1):e12525. doi: 10.1111/cpr.12525 (PMC6430464; doi:10.1111/cpr.12525)
Supplement: Supplementary file 1 [file CPR-52-e12525-s001.docx]

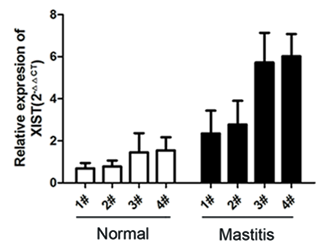


Fig. S**1. Expression levels of XIST in normal and mastitic samples of bovine mammary glands.** XIST genic expression levels in normal and mastitic tissues of bovine mammary glands (n=4) were analyzed by RT-qPCR.


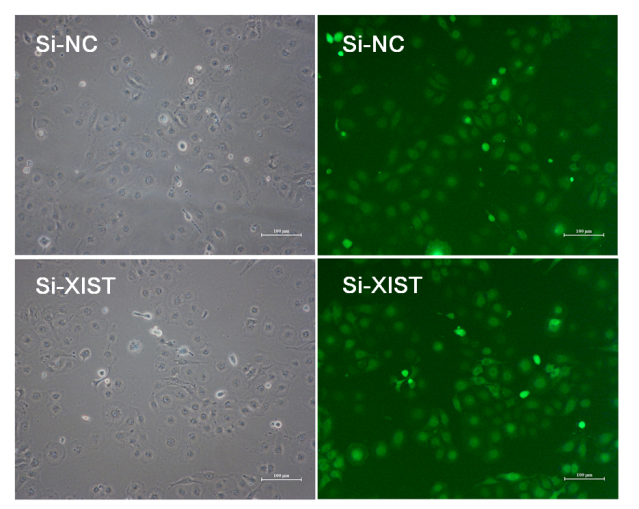


**Fig. S2. Transfection efficiency of the siRNAs.** To examine the transfection efficiency of Si-XIST and Si-NC, the MAC-T cells were transfected with FAM-labeled Si-XIST and Si-NC using Lipofectamine 2000 reagent, respectively. FAM-labeled siRNAs (Green) was observed in MAC-T cells by fluorescence microscopy. Abbreviations: Si-NC, a negative control siRNA; Si-XIST, an siRNA targeting bovine XIST.

**Table S1. PCR primers used in the present study**

| Gene name Forward Primers (5′-3′) Reverse Primers (5′-3′) |
| --- |
| GAPDH ACGGCACAGTCAAGGCAGA GTGATGGCGTGGACAGTGG  XIST GGCTAGAGGGTTGGTTAGGC CCTTTGCCTGTCGCATTATT |
| TNF-α GTTCTCCCCATGACACCACCTG GGGAGAAGAGAGTCAGACAGGC |
| IL-1β GCCTACGCACATGTCTTCCA GCAGAACACCACTTCTCGGT |
| IL-6 ACAGCTATGAACTCCCGCTT TCGACCATGCGCTTAATGAGA |
| NLRP3 TCTGCCATTCAGGTGTGGAC TTCACCAAGCAGAAGGACCA |
| Caspase-1 GGGATGGTATGGGTAAACGA TCCTTCTCTATGTGGGCTTTC  ASC GAAGACAGCCAAGCCAGGAC TGCTCCTCTGTCAGGACCTTC |
